# Freedom to think: the need for thorough assessment and treatment of gender dysphoric children

**DOI:** 10.1192/bjb.2020.72

**Published:** 2021-10

**Authors:** Marcus Evans

**Affiliations:** 1 Psychoanalyst, London, UK

**Keywords:** Psychiatry and law, consent and capacity, individual psychotherapy, clinical governance, comorbidity

## Abstract

Referrals (particularly natal female) to gender identity clinics have increased significantly in recent years. Understanding the reasons for this increase, and how to respond, is hampered by a politically charged debate regarding gender identity. This article starts with a discussion of the so-called ‘affirmative approach’ to gender dysphoria and considers the implications of the Memorandum of Understanding on conversion therapy. I then say something about the relationship between gender dysphoria and the developmental problems that are characteristic of adolescence. Finally, I outline what changes to the current approach are needed to do our best to ensure these patients receive the appropriate treatment.

There has been a 3264% rise in referrals to the national gender identity service at the Tavistock and Portman NHS Trust in London over the past 10 years (from 77 in 2009–2010 to 2590 in 2018–2019).^1^ The profile of referrals has also undergone a major transformation: we have seen a reversal of the gender ratio from two-thirds male:female to two-thirds female:male, with a recently described clinical phenomenon of as yet uncertain diagnostic significance making up a substantial proportion. This gender dysphoria of recent onset among adolescents (sometimes termed ‘recent-onset gender dysphoria' or ROGD, ‘rapid-onset adolescent dysphoria’^2^ or ‘adolescent-onset transgender history’^3^) lacks an agreed name or established diagnostic criteria, but its emergence has been documented by a number of gender clinics worldwide.^4^ Bernadette Wren, the then associate director of the Tavistock and Portman NHS Foundation Trust’s Gender Identity Development Service (GIDS), gave evidence to a House of Commons select committee in which she summarised the GIDS intake in the following terms: ‘many of the young people, and increasing numbers of them, have had a gender-uncontentious childhood, if you like, and it is only when they come into puberty and post-puberty that they begin to question. That now represents a substantial proportion of our group’.^5^ We have very little understanding of what underlies these changes, and indeed of the understanding of this whole area is extremely limited and it is particularly important to examine it from different perspectives. This is very difficult in the current environment, as the necessary debate and discussion is continually being closed down, either through individuals being prevented from expressing their views or being self-censoring through fear of the accusation of ‘transphobia’.

## The ‘affirmative approach’

The affirmative approach to gender dysphoria appears to have been adopted by the majority of NHS and privately provided children's services in the UK. Again, Bernadette Wren stated in the House of Commons: ‘I work in a service where a lot of the young people – and anybody who wants it – have physical intervention. We have no record of turning people down for physical intervention’.^5^ This approach endorses the child's belief that they were born in the wrong body and practitioners are required to support the child's self-identification. Commenting on the decision of the American Academy of Paediatrics (AAP), Cantor says ‘Although almost all clinics and professional associations in the world use what's called the “watchful waiting approach” to helping gender diverse children, the AAP statement instead rejected that consensus, endorsing gender affirmation as the only acceptable approach’.^6^ This is despite research findings which strongly suggest that most of these cases would eventually desist if left untreated.^7,8^ The ‘affirmative approach’ risks sending children down a path towards concrete and sometimes irreversible medical interventions for what is in very many cases a psychological problem. This approach, in my view, is driven by political ideology rather than clinical need and inhibits the clinician's curiosity and freedom to explore a child's underlying belief systems and motivations. The ‘affirmation approach’ looks narrowly at a problem in only one area of psychological functioning, as if one part of the individual could be isolated from other areas of the personality, so ignoring the complex relationship between the overt symptomatic picture and trauma, social anxieties and even the relatively normal turbulence of adolescence.

## The Memorandum of Understanding on Conversion

The Memorandum of Understanding on Conversion, which many healthcare professionals have signed, purports to protect the patient from conversion therapy. The Royal College of Psychiatrists signed the first version of the Memorandum (in 2015)^9^ as it referred to homosexuality but declined to sign the second Memorandum (2017)^10^ as the definition had been expanded to include transgender individuals. The Memorandum is very often interpreted as obstructing the clinician's freedom to examine and explore the various pathways that have led to gender dysphoria, but, somewhat surprisingly, when one reads the document one discovers an acknowledgment that the therapist or healthcare professionals’ job is to help the patient discover and come to terms with who they really are:

‘For people who are unhappy about their sexual orientation *or their transgender status* [my italics], there may be grounds for exploring therapeutic options to help them live more comfortably with it, reduce their distress and reach a greater degree of self-acceptance’.^11^

This Memorandum implies that there is a fixed category called ‘transgender’ which, like eye colour, is simply a given that need not be thought about or understood. Children's sexual orientation and gender identity are formed out of a complex developmental process that involves an interaction between their body, their mind and society at large. Sexual identity and gender identity are developmental processes that evolve as the individual goes through the different life stages.

The Memorandum is, in my view, symptomatic of the way that political agendas have influenced this area of clinical practice. We do not just accept/affirm a patient with anorexia when, although she weighs 45 kg, she thinks she is overweight and needs to diet more carefully. Instead, we take it as our duty to try to understand what it is that is driving that belief while persuading her that she needs to eat.

## Children with complex problems

There is a growing body of knowledge that connects the development of gender dysphoria with psychological factors.^12–17^ A group of parents whose children were treated at the Tavistock and Portman NHS Foundation Trust's Gender Identity Development Service (GIDS) in London wrote to the trust's board. In their letter they express deep concern that children with no long history of gender dysphoria, who were on the autism spectrum or suffered from social anxiety adjustment disorders were, with very insufficient investigation, diagnosed as transgender. They believed that the GIDS adopted a superficial approach that was in danger of colluding with the child's belief that all their problems will be solved if only they could change gender.

The parents wrote to *The Guardian*, which reported on Saturday 3 November 2018 that children were ‘fast-tracking’ young people into life-altering decisions without fully assessing their personal histories. Their letter stated that:^18^

‘the GIDS team is being asked to engage with and assess complex and difficult cases within a highly constrained time frame’.

They also believed that their children had been indoctrinated as a result of online websites that recruited the child into membership of the trans community.

## Research

One needs to be very cautious about recommending medical and surgical interventions that place a lifelong burden of treatment on patients. We know little about their effectiveness (there have been no long-term follow-up studies). Carl Heneghan, Oxford University's Professor of Evidence-Based Medicine and Editor-in-Chief of the journal *BMJ Evidence-Based Medicine*, has called the puberty blocker treatment an ‘unregulated live experiment on children’.^19^ *Research Digest*, published by the British Psychological Society, reported on an Australian review which concluded that the current medical approach is based on extremely limited evidence.^20^ The Royal Society of General Practitioners has drawn attention to ‘the significant lack of evidence for treatments and interventions which […] is a major issue facing this area’.^20^ Professor Robert Winston (Lord Winston) of Imperial College London has expressed concern about medically transitioning young people without having ‘really defined what is really wrong: what is the cause for people seeking gender reassignment? Until we define the problem, I think we're going to have a very blunderbuss problem’. Winston also highlights how often medical transition may not meet the expectations of patients:

‘He said 40 per cent of people who undergo vaginal reconstruction surgery experience complications as a result, and many need further surgery, and 23 per cent of people who have their breasts removed “feel uncomfortable with what they've done”. He added: “What I've been seeing in a fertility clinic are the long-term results of often very unhappy people who now feel quite badly damaged. “One has to consider when you're doing any kind of medicine where you're trying to do good not harm, and looking at the long-term effects of what you might be doing, and for me that is really a very important warning sign.” He added that the long-term effects of taking hormones “are likely to affect reproductive function”.’^21^

Even GIDS's own senior psychologist Bernadette Wren has mused: ‘Of course, you have to think that in another generation we will have done something which is not regarded as having been wise.’^22^ Professor Donal O'Shea, an endocrinologist, has been highly critical of the World Professional Association for Transgender Health's (WPATH's) Standards of Care, which inform NHS England's guidelines: ‘Aligning with them would result in significant harm accruing to those with gender confusion’. His colleague, psychiatrist Dr Paul Moran, considers that the WPATH guidelines ‘are clinically unsafe, and unsuitable for use in a public healthcare gender clinic.’^23^

## Patients that regret treatment

An increasing number of ‘regretters’ or ‘detransitioners’ are speaking out on social media and at conferences to argue they have been let down by mental health services that have failed to assess their psychological problems before prescribing medical treatments such as puberty blockers and cross-sex hormones, or surgery as treatment for their gender dysphoria. A number of clinicians have called for research into desistance, detransition and regret among gender dysphoric adolescents. The US National Institutes of Health (NIH) Sexual & Gender Minority Research Office (SGMRO) recently named detransition in its report outlining scientific research gap areas in the field of sexual and gender minority health.^24^ The 8th edition of WPATH's Standards of Care will include a section on detransitioning.^25^

Sky News reported on Saturday 5 October 2019 that Charlie Evans, who has detransitioned, has set up a charity to help others in a similar situation and has been contacted by hundreds of people seeking advice.^26^

During the 1980s, I led a parasuicide service in King's College Hospital, London, and treated a number of individuals who had self-harmed or attempted suicide after gender reassignment surgery. These patients had a history of serious and enduring mental illness and/or a personality disorder. Having developed a late-onset gender dysphoria, they were often angry at the loss of their biological sexual functioning and aggrieved with psychiatric services, which they felt had failed to examine their motivations for requesting reassignment surgery and/or to adequately investigate their psychological difficulties. A common theme in their presentations was a belief that physical treatments would remove or resolve aspects of themselves that caused them psychic pain. When the medical intervention failed to remove these psychological problems, the disappointment led to an escalation of self-harm and suicidal ideation, as resentment and hatred towards themselves were acted out in relation to their bodies.

## Informed consent

David Bell was approached by a large number of clinicians who had very serious ethical concerns about the service. His report was presented to the Tavistock and Portman NHS Foundation Trust board. In his report he wrote: ‘This is a highly complex and difficult area which appears at times to be treated superficially’. *The Times* (8 April 2019) quotes an anonymous clinician from the GIDS as saying: ‘It was regarded as taboo to discuss the impact of medical intervention on later sexual functioning in such a young cohort’.^27^

In the National Health Service (NHS), clinicians are usually required by law to discuss the potential negative effects of any treatment. However, for reasons that are not clear, the treatment for gender dysphoria has evolved operating outside the usual medical/professional practice. Children are signing up for treatments that permanently modify their bodies, but they may not fully understand the life-long consequences of their decision or acknowledge the potential risks and uncertainties of treatment. Their ability to provide informed consent has been questioned, including by some clinicians working in gender clinics.^28–30^ Are children of 12 and under really aware of what it will mean to become an infertile adult, who cannot have an orgasm and has to remain a patient dependent on hormones and medical care for the rest of their lives? Do girls know what it will mean in the future to have to undergo hysterectomy to avoid vaginal atrophy? It is also important to discuss openly that, although patients may decide in the long term to transition, they cannot eradicate the biological realities of their natal sex and will have to find some way of living with the losses involved. A full assessment and psychological engagement over a prolonged period can help the individual think through the social, psychological and biological implications of the medical interventions.

## Comprehensive assessment

A thorough assessment process involves two parts. First, an extended open-minded psychotherapeutic approach has the capacity to create the conditions whereby the factors, conscious and less conscious, that have led to the presentation can be understood (it needs to be borne in mind that a large number of individuals present with ‘rapid-onset gender dysphoria’, suggesting underlying triggering factors). An understanding of the family and social context will, of course, be critical. This difficult psychological work needs to be carried out by experienced mature and sensitive clinicians, as it can easily be felt to be threatening, especially where the individual presents with strongly held convictions – for example many believe that only a change in physical sexual identity can bring them the relief they need. There is considerable evidence of children and adolescents changing their minds if given enough time and space to explore things. Second, it is clearly vital that consent be fully explored. For example it will be important to gauge how much understanding the individual has of the implications of medical and surgical treatment. If an individual has no concern at all about the prospect and outcomes, this lack of concern should be thought of as *a symptom* that needs to be investigated and understood, rather than being treated superficially as a positive indication of their motivation. Unfortunately this kind of superficial approach is not uncommon. One needs to be able to empathise deeply with the individual's confusion, distress and mental pain, yet maintain adequate separation in order to be able to resist the pressure to join the patient in their view that active medical rather than psychological intervention is the only solution that can be even considered.

We must not forget our ordinary understanding of adolescence as a time of turmoil and considerable psychic pain as individuals have to come to terms with who they are, their strengths, weaknesses and limitations. Much of this, of course, centres on coming to terms with changes in the body and the new social roles that these changes demand. Gender services very often discuss only gender, with little mention of the relation with the changing sexual body. One young woman in my clinic, who was on a path to transitioning and then changed her mind, reported that there was no discussion of any biological realities in the pro-trans groups: ‘Lots of talk about gender politics and none about the physical realities involved in transitioning’. The majority of children prescribed puberty blockers go on to take cross-sex hormones.

## Gender conflicts are a normal part of development

We also need to bear in mind that adolescence is a time of experimentation that inevitably stirs up all sorts of conscious and unconscious confusions, doubts and conflicts which drive individuals to manage the anxiety and psychic pain through the use of powerful psychological defences such as denial, projection and splitting. When the child or adolescent is in danger of being overwhelmed there will be a tendency to focus on a fixed solution to deal with the most pressing concern, particularly the unbearable pain of confusion. The experience of being dislocated from one's body, which is changing rapidly in many ways, is not uncommon in adolescence. (This is perhaps one element of Kafka's classic tale *Metamorphosis* (1915) of a man who wakes up as a monstrous insect.) These feelings may be dealt with by premature foreclosure: ‘I am not the gender of the biology I was born with; I am the other’ – a statement that any experienced and mature clinician would resist through trying to create the conditions where confusion and psychic pain can be more tolerated. One of the central developmental tasks of adolescence is to come to terms with all sorts of realities, providing the basis for an integration of body and mind.

A political, rights-based approach to the treatment of children is at risk of forcing these complex psychological needs into the background. Pro-transitioning websites encourage children to view anyone who puts a different view, including parents, as suspect/the enemy; to self-diagnose and view the taking on of a trans identity as a wide-ranging solution to all their problems; to learn a script/obtain online tutoring so that the clinician who carries out the assessment will came to the ‘right’ conclusion, i.e. medical referral for transitioning. The so-called ‘affirmative approach’ persuades schools and others to accept unquestioningly the child's claims. Clinicians work in a take out zone where question is not welcomed. These various forces combine to ensure that these children very often get an assessment that is nowhere near adequate.

This radical disconnection of gender dysphoria from its potential roots in psychological disorder is fiercely promoted by pro-trans lobbies, who brand clinicians as ‘transphobic’ if they insist on a thorough psychological assessment of young people seeking to transgender/transition. That is, clinicians who are trying to protect the child from embarking prematurely on irreversible treatment are rebranded as a malign influence getting in the way of what the child ‘needs’.

In 2019, Dagny, a young woman who later realised her mistake and seeks to live again as her natal sex (a ‘de-transitioner’) published an article about her experience of transitioning. She highlights the influence of the online site Tumblr and gives a very good description of the ways in which she internalised the ideals of the website:^11^

‘One of these unhealthy beliefs I held was the belief that if you have gender dysphoria, you must transition. And anyone that appeared to stand in my way was a transphobe – an alt-right bigot.’

De-transitioners often describe being ostracised by the pro-trans group when they started to express doubts or question the treatment. Dagny writes that she became a different person when she started using Tumblr:

‘My online experience, having been affected by that level of groupthink, that level of moral policing and the constant implicit threats of social exposure and ostracisation made me an intensely internal and anxious person. It made me paranoid about the motives of people around me – I saw my parents as bigots because Tumblr told me to; because they held out for so long to prevent me from starting hormones.’

Children can also get online tutoring on how to get past the assessment process. *The Times* of 16 February 2019 also quoted David Bell as saying that they ‘have learnt through online resources [or] coaching from parents or peers exactly what to say in order to get the results they want’.^27^

Many parents have expressed concern that school counsellors and child and adolescent mental health services are adopting an unquestioning gender-affirmative approach. They describe how, once children announced that they believed they were the wrong sex, practitioners immediately endorsed this belief, often after only one meeting. Politically driven proposals proclaim the right of the child to define their own identity. But this denies the fact that identity is developed in relation to internal and external realities, both of which remain outside the individual's control. We do not control our biological inheritance and we cannot have complete control over the way we are seen by others.

## Political pressure on institutions and research

The extraordinary grip of powerful trans lobbies is having the effect of silencing clinicians who fear them. Television producers and journalists continually report that, although clinicians at GIDS are willing to speak in confidence to them about their reservations of treatment in these areas, they shy away from being named for fear of the consequences – being branded a transphobic bigot. Some fear disciplinary action being taken against them by their trust. Kenneth Zucker, a well-known researcher and clinical lead of the Child, Youth and Family Gender Identity Clinic in Toronto, was sacked from his post after being accused of conducting ‘conversion therapy’. The centre had a policy of first trying to help the individuals deepen their understanding of themselves before recommending medical interventions. The investigation subsequently completely exonerated Zucker.^31^ James Caspian, a psychotherapist with considerable experience of working with transgender patients, has described his sudden realisation of the increasing number of patients who regretted the sexual reassignment they had undertaken. In 2019, he wrote that he had been contacted by more than 50 patients in the preceding 2 years. However, his proposal to carry out a formal research project to investigate this phenomenon was rejected by his university department for fear of a backlash.^32^ In 2018, Lisa Littman described the insights of parents whose adolescent children had recently adopted a transgender identity – a phenomenon she provisionally labelled ‘rapid-onset gender dysphoria’.^33^ Littman's paper prompted huge controversy: *The Guardian* called her work ‘a poisonous lie used to discredit trans people’ and the result was that the paper was withdrawn, only to be subsequently republished with only very modest revisions.^34^

It is thus clear that this politically driven culture interferes with the freedom of thought necessary to work with these very troubled children and adolescents. It ceases to be possible for them to be assessed with an open mind, as individuals with their own unique difficulties and instead they become political symbols, actors in a wider ideological conflict – prejudice – and this is causing very serious damage.

## Conclusions

The fantasy that the body can be rapidly sculpted as a way of being rid of profound psychological problems needs to come under much closer scrutiny. There is a great reluctance to even consider that the difficulties can be understood, at least sometimes, through the lens of body dysmorphia, where the individual becomes obsessed with a perceived physical flaw. Plastic surgeons are very familiar with patients who seek surgery to erase a psychological difficulty and refer these individuals accordingly. Medical and surgical interventions in those with gender dysphoria very often leave the underlying problems completely unaddressed. It is, of course, not the case that surgical interventions can remove all evidence of natal sex – which remains as a source of persecution, a constant reminder of the continued existence of an unwanted aspect of the self. Individuals need help and support in coming to terms with who they are, as part of the maturational process. However, patients often put enormous pressure on family, schools and clinical services to join with them in the belief that to transition to the ‘ideal’ body, i.e. to eradicate unwanted aspects of their body, is the only solution to their problems. Perelberg makes the point that, if the family or clinical service accepts this without sufficient question, then there is a ‘confusion of registers’, i.e. the patient acts as if they are convinced that a problem of self-representation existing in the mind can be cured by concretely treating the body. The cost is that the individual is dissociated from their own body, treating it like a mannequin rather than a part of the self with anxieties, feelings and confusions.^35^

Whatever decisions are made regarding medical treatment, a thorough psychotherapeutic and psychiatric assessment is essential to enable us to help these vulnerable young people, their families and their clinical teams make informed decisions. It is a process of opening up a dialogue with the individual about their motives, beliefs, the issues they are struggling with – and, crucially, trying to understand the complex role of gender identity in their more global functioning. A clinician has a duty to protect and this cannot be honoured without a thorough understanding of who the child is and how they arrived at the place they are.

## Recommendations

Clinicians and patients need a service that is independent and protected from intrusions by pressure groups to force a rigid ‘one size fits all’ affirmative approach to gender dysphoria. NHS gender identity services have been functioning as if acting outside the ordinary requirement of good medical and psychiatric practice. The accusation of transphobia serves to shut down thoughtful enquiry and has been remarkably successful. As a result, the very thing that is most needed to protect children from harm is lost. This rapidly expanding and poorly understood phenomenon requires a new regulator tasked with appropriate oversight of gender identity services to ensure a more clinically rigorous, balanced and ethical approach to this complex area. Perhaps the Human Fertilisation and Embryology Authority (HFEA) developed to address ethical concerns in a rapidly expanding new field can provide the right kind of model.

## About the author

**Marcus Evans** is a former Head of Nursing and Associate Clinic Director of the Adult and Adolescent Departments, Tavistock and Portman NHS Foundation Trust, London. He is now a psychoanalyst in private practice and author of *Making Room for Madness in Mental Health: The Psychoanalytic Understanding of Psychotic Communication*.

## Supplementary material

For supplementary material accompanying this paper visit http://dx.doi.org/10.1192/bjb.2020.72. click here to view supplementary material
