# Freedom to think: the need for thorough assessment and treatment of gender dysphoric children – CORRIGENDUM

**DOI:** 10.1192/bjb.2020.124

**Published:** 2021-10

**Authors:** Marcus Evans

## Editor's note

The article ‘Freedom to think: the need for thorough assessment and treatment of gender dysphoric children’ was published online on 21 July 2020. In response, the *BJPsych Bulletin* received a number of letters both for and against the paper. The letters of complaint raised several similar issues, which were presented to the author for response. At our request, the author has addressed some omissions in the original article, particularly in relation to a declaration of interest of which we had been unaware. Also, some clarifications and additional references have been added to support a number of statements. An updated article is being published and we are publishing this corrigendum outlining the changes made – the original article remains available, as supplementary material to the updated article. We are keen to pursue an informed, transparent debate via the eLetters section, in line with the journal's eLetters policy. See also my editorial ‘Publishing controversy’ (https://doi.org/10.1192/bjb.2020.127).

**Norman Poole,** Editor of the *BJPsych Bulletin*

## Amendments and clarifications, by Marcus Evans

The opening sentences ‘There has been a 400% rise in referrals to the national gender identity service at the Tavistock and Portman NHS Trust in London over the past 5 years. The profile of referrals has also undergone a major transformation: we have seen a reversal of the gender ratio from two-thirds male:female to two thirds female:male, with a new diagnostic category, ‘recent-onset gender dysphoria’, making up a substantial proportion.” should read:

‘There has been a 3264% rise in referrals to the national gender identity service at the Tavistock and Portman NHS Trust in London over the past 10 years (from 77 in 2009–2010 to 2590 in 2018–2019).^c1^ The profile of referrals has also undergone a major transformation: we have seen a reversal of the gender ratio from two-thirds male:female to two-thirds female:male, with a recently described clinical phenomenon of as yet uncertain diagnostic significance making up a substantial proportion. This gender dysphoria of recent onset among adolescents (sometimes termed ‘recent-onset gender dysphoria’ or ROGD, ‘rapid-onset adolescent dysphoria’^c2^ or ‘adolescent-onset transgender history^c3^) lacks an agreed name or established diagnostic criteria, but its emergence has been documented by a number of gender clinics worldwide.^c4^’

The following should then be added:

‘Bernadette Wren, the then associate director of the Tavistock and Portman NHS Foundation Trust's Gender Identity Development Service (GIDS), gave evidence to a House of Commons select committee in which she summarised the GIDS intake in the following terms: “many of the young people, and increasing numbers of them, have had a gender-uncontentious childhood, if you like, and it is only when they come into puberty and post-puberty that they begin to question. That now represents a substantial proportion of our group”.^c5^’

The sentence ‘The affirmative approach to gender dysphoria has been adopted by the majority of children's services in the UK.’ should read:

‘The affirmative approach to gender dysphoria appears to have been adopted by the majority of NHS and privately provided children's services in the UK. Again, Bernadette Wren stated in the House of Commons: “I work in a service where a lot of the young people – and anybody who wants it – have physical intervention. We have no record of turning people down for physical intervention”.^c5^’

The section ‘Cantor says “Although almost all clinics and professional associations in the world use what's called the ‘watchful waiting approach’ to helping gender diverse children, the AAP statement instead rejected that consensus, endorsing gender affirmation as the only acceptable approach’.^1^ This is despite research findings which strongly suggests that most of these cases would eventually desist if left untreated.^2,3^’ should read:

‘Cantor says “Although almost all clinics and professional associations in the world use what's called the ‘watchful waiting approach’ to helping gender diverse children, the AAP statement instead rejected that consensus, endorsing gender affirmation as the only acceptable approach’.^c6^ This is despite research findings which strongly suggest that most of these cases would eventually desist if left untreated.^c7,^^c8^’

The sentence ‘The British Psychological Society's review of the literature found the current medical approach to be “well-intentioned advice [which] is based on extremely limited evidence”.^15^’ should read:

‘*Research Digest,* published by the British Psychological Society, reported on an Australian review which concluded that the current medical approach is based on extremely limited evidence.^15^’

The sentence ‘There is considerable evidence that children are signing up to treatments with long-term implications, with very little real understanding of the consequences for their future adult lives.’ should read:

‘Children are signing up for treatments that permanently modify their bodies, but they may not fully understand the life-long consequences of their decision or acknowledge the potential risks and uncertainties of treatment. Their ability to provide informed consent has been questioned, including by some clinicians working in gender clinics.^c9,^^c10,^^c11^’

The following sentence should be added after the first paragraph of the section ‘Patients that regret treatment’:

‘A number of clinicians have called for research into desistance, detransition and regret among gender dysphoric adolescents. The US National Institutes of Health (NIH) Sexual & Gender Minority Research Office (SGMRO) recently named detransition in its report outlining scientific research gap areas in the field of sexual and gender minority health.^c12^ The 8th edition of WPATH's Standards of Care will include a section on detransitioning.^c13^’

The following was omitted from the declaration of interest statement and should be included:

‘M.E. and his wife Sue Evans have provided witness statements for a UK judicial review examining whether minors are able to provide informed consent for gender-affirming treatments. M.E. has previously raised concerns about such treatments with the Board of Governors and the CEO of the Tavistock Trust. Sue Evans administrates the online pages for a crowd justice fund, which will be used to pay the legal fees of the judicial review. Neither M.E. nor Sue Evans has received or will receive any financial reward for participation in the case.’
